# Secondary cytomegalovirus infections: How much do we still not know? Comparison of children with symptomatic congenital cytomegalovirus born to mothers with primary and secondary infection

**DOI:** 10.3389/fped.2022.885926

**Published:** 2022-07-19

**Authors:** Fabiola Scaramuzzino, Michela Di Pastena, Sara Chiurchiu, Lorenza Romani, Maia De Luca, Giulia Lucignani, Donato Amodio, Annalisa Seccia, Pasquale Marsella, Teresa Grimaldi Capitello, Daniela Longo, Paolo Palma, Laura Lancella, Stefania Bernardi, Paolo Rossi, Francesca Ippolita Calo Carducci

**Affiliations:** ^1^Academic Department of Pediatrics, Bambino Gesù Children's Hospital, IRCCS, Rome, Italy; ^2^Clinical Psychology Unit, Department of Neuroscience, Bambino Gesù Children's Hospital, IRCCS, Rome, Italy; ^3^Immunological and Infectious Disease Unit, Academic Department of Paediatrics, Bambino Gesù Children's Hospital, IRCCS, Rome, Italy; ^4^Neuroradiology Unit, Imaging Department, Bambino Gesù Children's Hospital, IRCCS, Rome, Italy; ^5^Clinical Immunology and Vaccinology Unit, Academic Department of Paediatrics, Bambino Gesù Children's Hospital, IRCCS, Rome, Italy; ^6^Chair of Pediatrics, Department of Systems Medicine, University of Rome “Tor Vergata”, Rome, Italy; ^7^Audiology and Otosurgery Department, Bambino Gesù Children's Hospital, IRCCS, Rome, Italy

**Keywords:** congenital cytomegalovirus infection, CMV, secondary infection, pregnancy, symptoms, sequelae

## Abstract

Congenital cytomegalovirus (cCMV) infection can follow primary and secondary maternal infection. Growing evidence indicate that secondary maternal infections contribute to a much greater proportion of symptomatic cCMV than was previously thought. We performed a monocentric retrospective study of babies with cCMV evaluated from August 2004 to February 2021; we compared data of symptomatic children born to mothers with primary or secondary infection, both at birth and during follow up. Among the 145 babies with available data about maternal infection, 53 were classified as having symptomatic cCMV and were included in the study: 40 babies were born to mothers with primary infection and 13 babies were born to mothers with secondary infection. Analyzing data at birth, we found no statistical differences in the rate of clinical findings in the two groups, except for unilateral sensorineural hearing loss (SNHL) which was significantly more frequent in patients born to mother with secondary infection than in those born to mother with primary infection (46.2 vs. 17.5%, *P* = *0.037*). During follow up, we found a higher rate of many sequelae (tetraparesis, epilepsy, motor and speech delay, and unilateral SNHL) in the group of children born to mothers with secondary infection, with a statistical difference for tetraparesis and unilateral SNHL. Otherwise, only children born to mothers with primary infection presented bilateral SNHL both at birth and follow up. Our data suggest that the risk of symptomatic cCMV and long-term sequelae is similar in children born to mother with primary and secondary CMV infection; it is important to pay appropriate attention to seropositive mothers in order to prevent reinfection and to detect and possibly treat infected babies.

## Introduction

Congenital cytomegalovirus (cCMV) is the most common congenital infection affecting 0.5–2% of all live births, the leading non-genetic cause of SNHL, and a major cause of neurological disability (1).

Intrauterine transmission of CMV can follow primary or secondary maternal infection; the latter condition can be the result of either reactivation of latent CMV infection or reinfection with a different CMV strain during pregnancy (2).

This feature explains why the rate of cCMV infections increases with the seroprevalence of maternal populations, ranging from 0.3% in populations with 30% seroprevalence to approximately 2% in populations with 98% seroprevalence (3).

Transmission rates after primary maternal infection increase during pregnancy from 20–30% in the first trimester to 70% in the third trimester (4). Transmission rates by trimester due to maternal secondary infection are hard to assess because the diagnosis of this type of infection is difficult and consenquently it is not known how many women have reactivation or reinfection during pregnancy and how many congenital infections result from reactivation or reinfection (5).

In the past, symptomatic cCMV was thought to occur almost exclusively after primary maternal infection (6). Therefore, preventive measures for cCMV infection have been focused mainly on seronegative women. Nowadays, a growing body of evidence suggests that secondary maternal infection contributes to a much greater proportion of symptomatic cCMV than was previously thought (3, 5).

Thus, we performed a retrospective study analyzing data of symptomatic children with cCMV, comparing those born to mothers with primary infection with those born to mothers with secondary infection, both at birth and at follow-up.

## Materials and methods

### Study population

We performed a monocentric retrospective observational study of babies with cCMV evaluated in a tertiary care Pediatric Academic Hospital, without maternity ward, from August 2004 to February 2021. Children were referred to our center because of the presence of symptoms at birth consistent with cCMV or because of the serological evidence of maternal infection during pregnancy. We excluded from this study children with severe comorbidities and/or other congenital and perinatal infections.

Considering the retrospective nature of the analysis, the current study did not require the approval of the local ethics committee according to current legislation, but a notification was sent.

Data were retrospectively analyzed in line with personal data protection policies.

### Maternal infection

We collected data about the type of maternal infection. Primary maternal infection was defined in presence of seroconversion from negative to positive CMV IgG during pregnancy or presumed in presence of CMV IgM and a low CMV IgG-avidity. Secondary maternal infection was defined when previous infection was documented and presumed when at the beginning of the pregnancy women presented with positive IgG and negative IgM.

The time of maternal infection was estabilished according to the time of appearance of antibodies, without considering the lag beetween the infection and the antibody appearance. For secondary infection group the time of maternal infection was not assessed.

### Evaluation at birth

Diagnosis of cCMV infection was based either on detection of CMV DNA in urine and/or blood samples collected within the 21st day of life or on viral DNA detection on a Guthrie Card after the 21st day of life (7, 8).

We conducted the following investigations in all children with a confirmed diagnosis of cCMV: complete blood count, liver enzymes, conjugated bilirubin, renal function, cranial ultrasound scanning (CrUSS), abdominal ultrasound, audiological, ophthalmic and neurological assessment; we performed cerebral Magnetic Resonance Imaging (MRI) in babies with clinically detectable neurologic findings or CrUSS abnormalities until 2019; since January 2019 we started to perform cerebral MRI in all patients with cCMV and to consider isolated MRI abnormalities as sign of CNS involvement.

Babies were categorized at birth as “symptomatic” or “asymptomatic” according to the European Expert Consensus Statement on Diagnosis and Management of Congenital Cytomegalovirus (9).

We considered the nonspecific findings detected on CrUSS and cerebral MRI, such as lenticulostriated vasculopathy (LSV), as signs of CNS disease and consequently defined children as “symptomatic”; however, we did not treat children who presented those findings as the only sign of CMV disease (10).

### Treatment

We treated only babies with evidence of CNS disease, life-threatening disease, severe single-organ disease or multiorgan involvement; those patients received either intravenous ganciclovir (12 mg/kg/day divided into two daily doses) or oral valganciclovir (32 mg/kg/day divided into two daily doses) or a combination of both from the diagnosis of symptomatic disease, for a total of 6 weeks until 2015. Patients born after the publication of Kimberlin's study in 2015 received antiviral treatment for 6 months, in accordance with the evidence that emerged from the study (11).

To monitor for signs of toxicity, all treated babies underwent full blood count, liver function tests, urea, creatinine, and electrolytes weekly for the first 4 weeks and then at least monthly until completion of treatment course.

### Follow up

After discharge, all babies underwent a 6 years follow-up including pediatric clinical evaluation, audiologic, ophthalmic and neurodevelopmental assessment. Children with severe neurological disability (e.g., tetraparesis) were followed up longer.

All children were routinely screened for SNHL. Audiological evaluation was performed at birth and every 4 months until the age of 12 months, then twice a year during second and third year of life, thereafter by an annual audiometric surveillance. If a sensorineural hearing loss was detected, audiometric tests were recorded more frequently and longer.

Babies younger than 18 months of age were studied with objective tests: these included tympanometry, transient-evoked otoacoustic emissions (TEOAEs) and auditory brainstem response (ABR) assessment; from 2012 automated ABR (AABR) was introduced. Older patients underwent tympanometry, acoustic stapedius reflex threshold measurements, TEOAEs and puretone audiometry. The latter one was conducted with an age-specific test (behavioral observation audiometry in young children or visual reinforcement audiometry in older ones) and transducer (speakers for toddlers and earphones for more collaborating patients). Puretone audiometry was used to collect air and, if needed and possible, bone conduction thresholds. Older children with a suspicion of hearing loss, not compliant with puretone audiometry, underwent ABR testing for threshold under sedation.

Hearing loss was defined as absence of TEOAEs, an air conduction threshold >25 decibels hearing level (dB HL) on ABR, >35 dB HL on AABR, or >20 dB HL on age-specific puretone audiometry. Hearing loss was considered as sensorineural if the air-bone gap was <10 dB.

Neurodevelopmental assessment was performed using Bayley-III Scale to observe verbal, motor and fluid intelligent abilities until 3 years of age; WPPSI-III Scale to ages 4–7 years to parameterized general language, verbal, fluid intelligence and processing speed.

We considered similar constructs: Verbal Scale (Bayley-III)/General Language Scale (WPPSIIII); Cognitive Scale (Bayley-III)/Performance Scale (WPPSIIII); Motor Scale (Bayley-III)/Processing speed Scale (WPPSIIII). We considered a score below 85 IQ in each verbal, motor and cognitive index for each neurodevelopmental scale to be abnormal.

### Statistical analysis

We compared data of children born to mothers with primary infection with those born to mothers with secondary infection, both at birth and during follow up. Statistical analysis was performed using the Statistical Package for Social Science (SPSS). The Chi-square and the Fisher exact test were used to assess statistical significance of clinical data and outcome measures. *P*-values <0.05 were considered statistically significant.

## Results

We performed a monocentric retrospective observational study of babies with cCMV evaluated in our center from August 2004 to February 2021. In total, we identified 175 babies with cCMV:

- 118 born to mothers with primary infection (67.4 %)
- 27 born to mothers with secondary infection (15.4 %)
- 30, with unavailable data about maternal infection (17.1%)

We included in our study only babies with available data about maternal infection. Thus, 145 babies were considered in our study:

- 118 born to mothers with primary infection (81.4%)
- 27 born to mothers with secondary infection (18.6%)

The mean follow-up for children born to mothers with primary and secondary infection was 37.2 ± 20.5 months and 55 ± 48.7 months, respectively.

At birth 53 infants were classified as having a symptomatic cCMV infection: 40 babies born to mothers with primary infection and 13 babies born to mothers with secondary infection. No significant difference was found in the rate of symptomatic infection at birth between the two groups of children (33.9 vs 48.1%, *P* = *0165*).

In the group of symptomatic children born to mothers with primary infection, 22 were tested for CMV infection in the 1^st^ day of life, 4 in the 2^nd^, 1 in the 5^th^, 3 in the 8^th^, 2 in the 10^th^, 1 in the 11^th^, 1 in the 12^th^, 2 in the 13^th^, 2 in the 15^th^, 1 in the 17^th^, 1 in the 20^th^ day of life. CMV-PCR resulted positive on both blood and urine in 12 patients, on blood in 7 patients (not tested on urine), on urine in 21 patients.

In the group of symptomatic children born to mothers with secondary infection 1 was tested for CMV infection in the 1^st^ day of life, 1 in the 2^nd^, 2 in the 3^th^, 4 in the 10^th^, 2 in the 12^th^, 3 in the 15^th^ day of life. CMV-PCR resulted positive on both blood and urine in 2 patients, on blood in 5 patients (not tested on urine), on urine in 6 patients.

In the group of children born to mothers with primary infection, the time of maternal infection was mainly the first trimester of pregnancy (45% of cases); the percentage of infections acquired in subsequent trimesters was lower and decreasing (32.5% and 20%). Of the 18 women infected during the first trimester, 3 were diagnosed because of seroconversion, 15 because of positivity of IgM and low avidity IgG; of the 13 patients infected during the secondary trimester, 12 were diagnosed because of seroconversion, 1 because of positivity of IgM and low avidity IgG (tested at 22 weeks of gestational age); in all the 8 patients infected during the third trimester seroconversion was documented.

In the group of mothers with secondary infection, 8 were known to be CMV seropositive prior to current pregnancy and 5 presented at the beginning of pregnancy (6–10 weeks of gestational age) with positive IgG and negative IgM.

Table 1 shows the signs and symptoms presented at birth. We found only one symptom with a significant difference between the two groups: the rate of unilateral SNHL, which was most common in children born to mothers with secondary infection (46.2 vs. 17.5%, *P* = *0.037*).

**Table 1:** Signs and symptoms presented at birth by 53 symptomatic children divided according to the type of maternal CMV infection (primary or secondary).

	**Maternal primary infection** **(*****n** =* **40)**	**Maternal secondary infection** **(*****n** =* **13)**	***P***
IUGR	2 (5%)	1 (7.7%)	1
Congenital hydrocephalus	0	1 (7.7%)	0.245
SGA*	1 (2.5%)	1 (7.7%)	0.434
Prematurity	6 (15%)	2 (15.4%)	1
Apgar <7 at 1 min	2 (10%)	2 (15.4%)	0.249
Microcephaly**	2 (5%)	2 (15.4%)	0.249
Neurological signs	11 (27.5%)	6 (46.2%)	0.211
Chorioretinitis	0	1 (7.7%)	0.245
Thrombocytopenia	4 (10%)	1 (7.7%)	1
Anemia	1 (2.5%)	0	1
Splenomegaly	3 (7.5%)	0	0.567
Hypertransaminasemia	3 (7.5%)	3 (23%)	0.15
Hyperbilirubinemia	3 (7.5%)	0	0.567
Hepatomegaly	4 (10%)	0	0.561
Unilateral SNHL	(17.5%)	6 (46.2%)	0.037
Bilateral SNHL	4 (10%)	0	0.561
Abnormal CrUSS	20 (50%)	8 (61.5%)	0.469
Abnormal MRI	14/21 (66.7%)	3/6 (50%)	0.638

^*^SGA (Small for Gestational Age): defined as birthweight below the 10th percentile for gestational age at delivery.

^**^Microcephaly: defined as age-appropriate head circumference below two SD.

Only children born to mothers with primary infection had bilateral SNHL.

Regarding prenatal signs of fetal disease, 5% of children born to mothers with primary infection and 7.7% of children born to mothers with secondary infection presented intrauterine growth restriction (IUGR); one child born to mother with secondary infection showed a congenital hydrocephalus on fetal ultrasound later confirmed by fetal MRI.

Neurological signs, e.g. hypotone, hypertone and seizures, were presented by 27.5% of children born to mothers with primary infection and by 46.2% of children born to mothers with secondary infection.

In babies born to mothers with primary or secondary infection, abnormal CrUSS was found in 50% and 61.5%, respectively. In particular, we analized the rate of the following findings at CrUSS in both groups without finding any significant difference: calcification (15 vs. 7.7%, *P = 0.666*), cysts/pseudocysts (17.5 vs. 30%, *P = 0.432*), ventriculomegaly (2.5 vs. 15.4%, *P = 0.145*), white matter abnormalities (7.5 vs. 23, *P = 0.15*), LSV (17.5 vs. 30.8%, *P = 0.432*).

Table 2 shows the incidence of each sequela in the two groups of patients. We found a higher rate of many sequelae (tetraparesis, epilepsy, motor delay, speech delay, and unilateral SNHL) in the group of children born to mother with secondary infection, with a statistical difference for tetraparesis and unilateral SNHL; in particular, tetraparesis was presented by 30.8% of the children born to mothers with secondary infection but was not presented in the other group. Among the 5 children with unilateral SNHL born to mothers with primary infection, deafness was profound in all cases; among the 6 born to mothers with secondary infection, deafness was profound in 2 children and mild in 4 children.

**Table 2:** Outcome in 53 symptomatic patients divided according to the type of maternal CMV infection.

	**Maternal primary infection** **(*****n** =* **40)**	**Maternal secondary infection** **(*****n** =* **13)**	***P***
Tetraparesis	0	4 (30.8%)	0.002
Epilepsy	1 (2.5%)	1 (7.7%)	0.434
Motor delay	9 (22.5%)	5 (38.5%)	0.257
Speech delay	12 (30%)	7 (53.8%)	0.119
Cognitive delay	0	0	1
Any neurodevelopmental impairment	18 (45%)	7 (53,8%)	0.579
Unilateral SNHL	5 (12.5%)	6 (46.2%)	0.009
Bilateral SNHL	6 (15%)	0	0.317
Any sensorineural hearing impairment	11 (27.5%)	6 (46.2%)	0.306

The only sequela presented only by the group of children born to mothers with primary infection was bilateral SNHL: 6 babies presented bilateral and profound SNHL and required cochlear implantation.

Motor delay, speech delay and cognitive delay were observed with Bayley III and WPPSI III neurocognitive scale.

The neurocognitive sequela observed with Bayley III and WPPSI III scales in children born to mothers with primary infection showed: Linguistic-Verbal Scale Mean IQ 95 with a range score between IQ 60 and QI 120, speech delay in 30% of score cases; Mean Motor Scale-Processing speed IQ 96 with a range score between IQ 52 and IQ 118, motor delay in 22.5% of score cases. Mean Cognitive-Performance Scale IQ 105 with a range score between IQ 85 and IQ 115.

The neurocognitive sequela observed with Bayley III and WPPSI III scales in children born to mothers with secondary infection showed: Linguistic-Verbal Scale Mean IQ 95 with a range score between QI 60 and QI 120, speech delay in 53.8% of score cases; Mean Motor-Processing Speed Scale IQ 96 with a range score between 52–118, motor delay in 38.5% of score cases; Mean Cognitive-Performance Scale IQ 100.8 with a range score between IQ 85 and IQ 105.

We treated 57.5% of babies born to mothers with primary infection and 84.6% of babies born to mothers with secondary infection; no statistical difference in the rate of treated babies was found between the two group *(P* = *0.102)*.

Table 3 shows the outcome of symptomatic children born to mothers with primary infection divided by trimester of maternal infection. The majority of neurodevelopmental and auditory sequelae were found in babies born to mothers infected in the first trimester of pregnancy, who developed sequelae in 66.7% of cases. Nevertheless, three babies born to mothers infected at second trimester (23%) and one infected at third trimester (12.5%) reported severe sequelae.

**Table 3:** Outcome of symptomatic children born to mothers with primary infection divided according to trimester of infection.

**Trimester of maternal infection**	**Symptomatic babies for each trimester (total** ***n** =* **40)**	**Any sequela**	**Neurodevelopment impairment**	**Unilateral SNHL**	**Bilateral SNHL**
I	18 (45%)	12 (66.7%)	8 (44%)	2 (11%)	4 (22%)
II	13 (32.5%)	3 (23%)	1 (7.7%)	1 (7.7%)	2 (15%)
III	8 (20%)	1 (12.5%)	1 (12.5%)	1 (12.5%)	0
Unknown	1 (2.5%)	0	0	0	0

Regarding the incidence of sequelae in babies born to mothers with secondary infection, we have scanty data about the trimester of reactivation/reinfection thus it is not possible to draw any conclusion.

## Discussion

To date, we are not aware of the real incidence of secondary CMV infection during pregnancy. Thus, we do not know how many congenital infections result from this condition and what is the exact frequency and full spectrum of signs, symptoms and sequelae presented by children born to mothers with non-primary infection (12).

A population-based prediction model found that secondary infections are responsible for the majority of cCMV infections as well as CMV-related hearing loss (3).

A recent meta-analysis, including 879 children, indicated that symptomatic cCMV infection at birth is not associated with type of maternal infection and that the risk of long-term sequelae is similar in children born to mother with primary and non-primary CMV infection (5).

In this study, we compared data from symptomatic children born to mothers with primary infection with those born to mothers with secondary infection, both at birth and during long-term follow-up (37.2 and 55 months, respectively), assessing whether there were differences in the rate of signs and symptoms at birth, abnormal findings on neuroimaging, or sequelae at follow-up.

We found no significant differences in the rate of symptomatic children at birth between the two groups (33.9 vs. 48.1%, *P* = *0.165*). Giannattasio et al. described similar results: they found 46.2% symptomatic newborns to mother with primary infection and 60% symptomatic newborns to mother with secondary infection (13). They reported a significantly higher rate of neurological signs, chorioretinitis and thrombocytopenia in children born to mothers with secondary infection, whereas we found no statistical differences in the rate of clinical findings at birth in the two groups, with the only exception of SNHL which was significantly more common in children born to mothers with secondary infection. In our cohort, neurological signs were the most frequent of all signs/symptoms presented at birth in both groups, with a higher rate in the group of children born to mothers with secondary infection without reaching statistical significance.

Considering unilateral and bilateral SNHL together, the incidence in the two groups was similar; 27.5% children born to mothers with primary infection and 46.2% children born to mothers with secondary infection presented SNHL at birth. Consistently, Ross et al. reported in a cohort of 300 children, 124 born to mothers with either presumed or confirmed secondary infection and 176 born to mothers with either presumed or confirmed primary CMV infection, a similar rate for hearing loss in the two groups; they considered both symptomatic and asymptomatic children in their study (14).

Regarding neuroimaging, 50% of babies born to mothers with primary infection and 61.5% of those born to mothers with secondary infection had an abnormal finding at CrUSS. We found a higher incidence of cysts/pseudocysts, ventriculomegaly, white matter abnormalities and LSV in children born to mother with secondary infection; instead, calcifications were prevalent in the group of children born to mothers with primary infection. None of the findings had a significantly different rate in the two groups. Hadar et al. reported a significantly higher rate of abnormal finding at CrUSS in babies born to mothers with primary infection; however, in their cohort there was a much higher number of symptomatic children born to mothers with primary infection than children born to mothers with secondary infection (95 vs. 12) (15). In addition, the type of pathologic findings at CrUSS was not specified. In the cohort of patients described by Giannattasio et al. there was a similar rate of brain abnormalities in the group of children born to mothers with primary infection and those born to mothers with secondary infection (13).

MRI was performed in 20 babies born to mothers with primary infection and 6 babies born to mothers with secondary infection; the exam reported abnormal findings in 66.7% and 50% of those babies, respectively, without statistically significant differences.

During follow up, we found a similar rate of neurodevelopmental sequelae in the two groups, as previously reported by Puhakka et al. (13), Giannattasio et al. (16), and Coscia et al. (17). Nethertheless, analyzing each sequela separately (tetraparesis, epilepsy, motor and speech delay) we found a statistical difference for tetraparesis that was more frequent in children born to mothers with secondary infection (30 vs. 0%).

Considering audiological impairment, we found that the higher rate of unilateral SNHL in children born to mothers with secondary infection was maintained at follow-up; moreover, none of these children developed bilateral SNHL. In our cohort only children born to mothers with primary infection presented bilateral SNHL: 4 children at birth and 6 during follow-up. Consistently, Ross et al. reported in their cohort that fewer children in the secondary infection group had bilateral hearing loss compared with the primary infection group, and significantly fewer children born to mothers with preexisting seroimmunity had progression of their hearing loss compared with those born to mothers without prior immunity (14).

Considering that the likelihood of fetal harm is greater when CMV infection occurs early in pregnancy, we analyzed data from symptomatic newborns divided by trimester of maternal infection (18). We assessed the long-term outcome of symptomatic children born to mothers with primary infection, divided by trimester of maternal infection. As expected, we found that symptomatic children born to mothers who acquired the infection in the first trimester developed sequelae in 66.7% of cases. Surprisingly, we also found that 23% and 12.5% of the symptomatic children born to mothers infected during the second and the third trimester, respectively, reported severe sequelae at follow up. Our results differ from those reported by the meta-analysis of Chatzakis et al. They found that severe sequelae are common only when maternal infection occurs in the periconceptional period and in the first trimester (29% and 19%, respectively), decreasing to ~1% after this point (18).

The important difference in the rate of sequelae could be explained by the fact that our cohort is composed only by symptomatic infants, who more often have an unfavorable outcome. Moreover, the number of patients is too small to draw numerical conclusions. Nonetheless, we want to highlight that primary maternal infections acquired in the second and third trimesters of pregnancy can lead to long-term sequelae in children, although to a lesser extent than those acquired in the first one.

This retrospective study presents several limitations. First of all, the numbers on which comparison are performed are small and thus not conclusive. The proportion of symptomatic patients in the cohort is very high and the frequency and severity of sequelae in the symptomatic group is also higher than expected from the literature. This could be explained by the fact that many of those children were referred to a tertiary center because of the severity of symptoms. This selection bias particularly affects the group of children born to mothers with secondary infection; many of these children were tested at birth for CMV because of suggestive symptoms of infection, even without documented reinfection/reactivation of the virus during pregnancy.

Furthermore, the population included is extremely heterogeneous both because of the different criteria adopted over the years to define a “symptomatic” baby and the different treatment strategies applied over the years, which have a different impact on the long-term outcomes.

Thus, it is not possible to draw firm conclusions from this retrospective study regarding the overall, population-based frequency of disability or impairments due congenital CMV infection from primary or secondary maternal infection. Making those estimates and comparison requires prospective identification of maternal infections during pregnancy and screening newborns for congenital CMV infection.

Another limitation of this retrospective study is that follow up data where not complete for all patients; morover, serological maternal data were available from medical files and sera could not be retrived to perform new standardized test for this study.

In conclusion, our study aims to emphasize that cCMV infections due to secondary maternal infections can be as serious as those due to primary infections, both at birth and during follow up; the less attention often given to seropositive mothers can lead to missed diagnosis of congenital infections in children who may develop serious sequelae. Boppana et al. demonstrated that two-thirds of CMV infection in previously seropositive women were due to exogenous reinfection (19). Thus, increasing hygienic measures in those women may reduce the number of reinfection and, consequently, the number of cCMV. Moreover, paying more attention to seropositive women could allow to detect and possibly treat otherwise unrecognized infected babies.

## Data availability statement

The raw data supporting the conclusions of this article will be made available by the authors, without undue reservation.

## Ethics statement

Ethical review and approval was not required for the study on human participants in accordance with the local legislation and institutional requirements. Written informed consent for participation was not provided by the participants' legal guardians/next of kin because of the retrospective nature of the analysis, but a notification to the Ethics Committee was sent. Data were retrospectively analyzed in line with personal data protection policies.

## Author contributions

FS participated to the design of the study, analyzed data, interpreted results, and drafted the article. SB, LL, PP, and PR contributed to the revision of draft and interpretation of data. LR, SC, MDL, DA, DL, GL, AS, and PM were involved in the acquisition and analysis of data. MDP and TG performed and revised neurodevelopmental outcomes. FC conceived the study and revised the article critically for important intellectual content.

## Conflict of interest

The authors declare that the research was conducted in the absence of any commercial or financial relationships that could be construed as a potential conflict of interest.

## Publisher's note

All claims expressed in this article are solely those of the authors and do not necessarily represent those of their affiliated organizations, or those of the publisher, the editors and the reviewers. Any product that may be evaluated in this article, or claim that may be made by its manufacturer, is not guaranteed or endorsed by the publisher.
